# Medicinal Plants in Therapy: Antioxidant Activities

**DOI:** 10.1155/2016/7468524

**Published:** 2016-04-11

**Authors:** Mohamed A. Dkhil, Denis Delic, Hesham A. El Enshasy, Ahmed E. Abdel Moneim

**Affiliations:** ^1^Department of Zoology and Entomology, Faculty of Science, Helwan University, Ain Helwan, Cairo, Egypt; ^2^Department of Biology, Heinrich Heine University, Duesseldorf, Germany; ^3^Innovation and Products Development, Institute of Bioproduct Development, Universiti Teknologi, Johor, Malaysia; ^4^Biomedical Research Center, Health Sciences Technology Park, University of Granada, Granada, Spain

In view of the oxidative stress implicated in the pathophysiology of a wide variety of different diseases, a therapeutic strategy to elevate the antioxidant defenses of the body may be of assistance in protecting from different diseases and disorders. This special issue includes 19 articles that emphasize the importance of medicinal plants in augmenting oxidative stress in cancer, aging, inflammation, diabetes, and heavy metal toxicity.

Several original and review articles discuss the role of medicinal plants in oxidative stress participating in diseases. T. Esatbeyoglu et al. and H. Qiao et al. open the issue by presenting the antioxidant and anti-inflammatory properties of a stilbenoid-rich root extract of* Vitis vinifera* and polydatin extracted from* Polygonum cuspidatum* in protecting against hydrogen peroxide-induced DNA damage. Q. Wang et al. show the ability of bioactive peptides from* Angelica sinensis* to attenuate aging process in* Caenorhabditis elegans* through antioxidant activities independent of dietary restriction. A. C. F. Salgueiro et al. find that tea prepared from* Bauhinia forficata* can protect from diabetes-induced liver damage and N. Sarega et al. present interesting data on the ability of* Clinacanthus nutans *to attenuate hyperlipidemia-associated oxidative stress in rats. Two other research articles focus on the anticancer effects of caffeic acid phenethyl ester and cocoa beans. D. Bauer et al. discuss the ability of cocoa beans to inhibit cell proliferation, arrest cell cycle in different phases, and increase apoptosis in human lung carcinoma cells. T. K. Motawi et al. show that caffeic acid phenethyl ester augmented tamoxifen cytotoxicity via multiple mechanisms, providing a novel therapeutic approach for breast cancer treatment that can overcome resistance and lower toxicity. In addition, A. Curnow and S. J. Owen examine* Althea officinalis* and* Astragalus membranaceus* extracts against UVA-induced DNA damage in cultured human lung and skin fibroblasts. The authors found that both the plants protected from UVA-induced oxidative stress and DNA damage for a greater period of time than the commercial field-grown roots. H. Khan et al. examined the antiplasmodial effects of 11-O-galloylbergenin isolated from* Mallotus philippensis* and they found that the isolated compound is good enough for its potency and effectiveness against* Plasmodium falciparum*. Furthermore, F. V. S. de Araújo Pinho et al. and J. Coccimiglio et al. studied the potential activities of* Duguetia furfuracea* A. St.-Hil. and* Origanum vulgare*. Finally, H.-R. Choi et al. isolated phlorizin from* Eleutherococcus senticosus *and studied its potential on human keratinocytes and skin equivalents. In this study, the authors found that phlorizin can affect the proliferative potential of epidermal cells in part by microenvironment changes via miR135b downregulation and following increased expression of type IV collagen.

